# Pyridine-2,6-dicarboxaldehyde bis[(diphenylmethylidene)hydrazone]

**DOI:** 10.1107/S1600536811011238

**Published:** 2011-04-07

**Authors:** Florina Dumitru, Mihaela-Diana Şerb, Ulli Englert

**Affiliations:** a Faculty of Applied Chemistry and Materials Science, University Politehnica of Bucharest, Polizu 1, RO-011061 Bucharest, Romania; b Institut für Anorganische Chemie, RWTH Aachen, Landoltweg 1, 52074 Aachen, Germany

## Abstract

The title compound, C_33_H_25_N_5_, belongs to the family of pyridine-2,6-dicarboxaldehyde Schiff bases which possess a terdentate coordinating site (–N=C–C=N–C–C=N–) similar to terpyridine derivatives. The dihedral angles between pairs of terminal rings are 69.67 (9) and 66.23 (9)°. The shortest distance between the centroids of aromatic rings in neighbouring mol­ecules is 3.8080 (14) Å.

## Related literature

For compounds containing the (–N=C–C=N–C–C=N–) moiety in acyclic ligands, see: Vance *et al.* (1998 ▶); Albrecht *et al.* (2007 ▶) and in macrocyclic ligands, see: Haussmann *et al.* (2007 ▶); Plattner *et al.* (2002 ▶). For electrostatic inter­actions between the nitro­gen lone pairs, which determine the all-*trans* transoid solid-state configuration of the archetypal terpyridine ligand, see: Fallahpour (2003 ▶); Constable (2007 ▶).
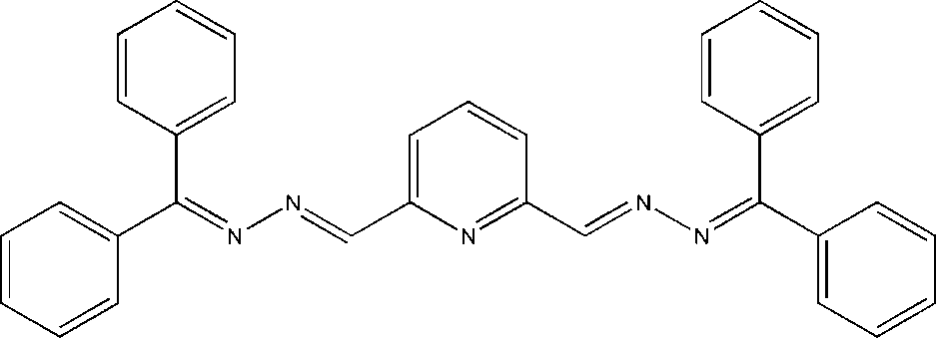


## Experimental

### 

#### Crystal data

C_33_H_25_N_5_
*M* *_r_* = 491.58
Monoclinic, 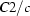

*a* = 23.702 (5) Å
*b* = 12.344 (3) Å
*c* = 18.758 (4) Å
β = 106.742 (4)°
*V* = 5255.3 (19) Å^3^
*Z* = 8
Mo *K*α radiation
μ = 0.08 mm^−1^
*T* = 100 K
0.40 × 0.17 × 0.08 mm

#### Data collection

Bruker SMART APEX CCD diffractometer
Absorption correction: multi-scan (*SADABS*; Bruker, 2004 ▶) *T*_min_ = 0.971, *T*_max_ = 0.994
30994 measured reflections
5391 independent reflections
3723 reflections with *I* > 2σ(*I*)
*R*_int_ = 0.090

#### Refinement

*R*[*F* ^2^ > 2σ(*F* ^2^)] = 0.048
*wR*(*F* ^2^) = 0.117
*S* = 1.04
5391 reflections
343 parameters
H-atom parameters constrained
Δρ_max_ = 0.21 e Å^−3^
Δρ_min_ = −0.21 e Å^−3^

### 

Data collection: *SMART* (Bruker, 2001 ▶); cell refinement: *SAINT-Plus* (Bruker, 1999 ▶); data reduction: *SAINT-Plus*; program(s) used to solve structure: *SHELXS97* (Sheldrick, 2008 ▶); program(s) used to refine structure: *SHELXL97* (Sheldrick, 2008 ▶); molecular graphics: *PLATON* (Spek, 2009 ▶); software used to prepare material for publication: *SHELXL97*.

## Supplementary Material

Crystal structure: contains datablocks global, I. DOI: 10.1107/S1600536811011238/bt5500sup1.cif

Structure factors: contains datablocks I. DOI: 10.1107/S1600536811011238/bt5500Isup2.hkl

Additional supplementary materials:  crystallographic information; 3D view; checkCIF report

## Figures and Tables

**Fig. 1.**
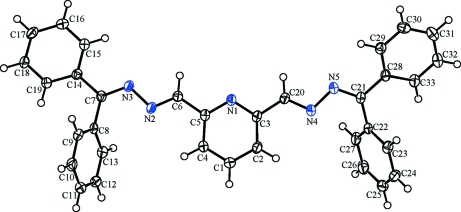
: PLATON (Spek, 2009) plot with displacement ellipsoids at 50% probability; H atoms are represented by spheres of arbitrary radius.

## supplementary crystallographic information

### Comment

The title compound is structurally related to terpyridyl (unsaturated nitrogen donor group incorporated into a tridentate frame) and offers a terpyridine-like coordination environment through a highly efficient and simple ligand synthesis. Analogously to terpyridine-ligands which exhibit all-*trans transoid* configurations about the interannular carbon-carbon bonds in order to minimize electrostatic interactions between the nitrogen lone pairs (Fallahpour, 2003; Constable, 2007), pyridine-2,6-dicarboxaldehydebis(benzophenone hydrazone) presents a *transoid* conformation for the (–N═C–C═N–C–C═N–) moiety wherein the lone-pair electrons of adjacent N atoms are directed away from each other. The dihedral angle formed by the phenyl rings attached to C7 is 69.67 (9)° and the dihedral angle formed by the phenyl rings attached to C21 is 66.23 (9)°. The shortest distance between the centroids of aromatic rings in neighbouring molecules amounts to 3.8080 (14) Å [Cg(1)-Cg(3)^i^; ring (1): N1-C3-C2-C1-C4-C5; ring (3): C14-C15-C16-C17-C18-C19; symmetry operator (i): 1-x, 1-y, 1-z].

### Experimental

pyridine-2,6-dicarboxaldehyde has been prepared according to the procedure of Vance *et al.* (1998) by oxidation of 2,6-pyridinemethanol with activated manganese(IV) dioxide. pyridine-2,6-dicarboxaldehydebis(benzophenone hydrazone) has been synthesized by condensation of one equivalent of pyridine-2,6-dicarboxaldehyde (0.135 g, 1 mmol) with two equivalents of benzophenone hydrazone (0.392 g, 2 mmol) in MeOH (40 ml) with stirring under reflux for 2 h. After solvent evaporation, the resulting crude material was recrystallized from acetonitrile to give the title compound as yellow crystals. ^1^H-NMR (400 Hz, CD_3_CN, p.p.m.): δ 8.413 (s, 2H, CH═N), 7.717 (br, 3H, H^py^), 7.692 (m, 2H, H^ar^), 7.674–7.671 (d, 2H, H^ar^), 7.511–7.498 (t, 1H, H^ar^), 7.489–7.482 (t, 1H, H^ar^), 7.460–7.454 (d, 5H, H^ar^), 7.446–7.442 (d, 5H, H^ar^), 7.325–7.320 (d, 2H, H^ar^), 7.312–7.301 (d, 2H, H^ar^).

### Refinement

H atoms  were  introduced in their idealized positions with C—H 0.95 Å, *U*_iso_(H) = 1.2*U*_eq_(C).

### Crystal data

**Table:**

C_33_H_25_N_5_	*F*(000) = 2064
*M**_r_* = 491.58	*D*_x_ = 1.243 Mg m^−^^3^
Monoclinic, *C*2/*c*	Mo *K*α radiation, λ = 0.71073 Å
Hall symbol: -C 2yc	Cell parameters from 1380 reflections
*a* = 23.702 (5) Å	θ = 2.3–20.1°
*b* = 12.344 (3) Å	µ = 0.08 mm^−^^1^
*c* = 18.758 (4) Å	*T* = 100 K
β = 106.742 (4)°	Fragment, yellow
*V* = 5255.3 (19) Å^3^	0.40 × 0.17 × 0.08 mm
*Z* = 8	

### Data collection

**Table:**

Bruker SMART APEX CCD diffractometer	5391 independent reflections
Radiation source: fine-focus sealed tube	3723 reflections with *I* > 2σ(*I*)
graphite	*R*_int_ = 0.090
ω scans	θ_max_ = 26.4°, θ_min_ = 2.3°
Absorption correction: multi-scan (*SADABS*; Bruker, 2004)	*h* = −29→29
*T*_min_ = 0.971, *T*_max_ = 0.994	*k* = −15→15
30994 measured reflections	*l* = −23→23

### Refinement

**Table:**

Refinement on *F*^2^	Primary atom site location: structure-invariant direct methods
Least-squares matrix: full	Secondary atom site location: difference Fourier map
*R*[*F*^2^ > 2σ(*F*^2^)] = 0.048	Hydrogen site location: inferred from neighbouring sites
*wR*(*F*^2^) = 0.117	H-atom parameters constrained
*S* = 1.04	*w* = 1/[σ^2^(*F*_o_^2^) + (0.0352*P*)^2^ + 2.9488*P*] where *P* = (*F*_o_^2^ + 2*F*_c_^2^)/3
5391 reflections	(Δ/σ)_max_ = 0.001
343 parameters	Δρ_max_ = 0.21 e Å^−^^3^
0 restraints	Δρ_min_ = −0.21 e Å^−^^3^

### Special details

**Table:**

Geometry. All e.s.d.'s (except the e.s.d. in the dihedral angle between two l.s. planes) are estimated using the full covariance matrix. The cell e.s.d.'s are taken into account individually in the estimation of e.s.d.'s in distances, angles and torsion angles; correlations between e.s.d.'s in cell parameters are only used when they are defined by crystal symmetry. An approximate (isotropic) treatment of cell e.s.d.'s is used for estimating e.s.d.'s involving l.s. planes.
Refinement. Refinement of *F*^2^ against ALL reflections. The weighted *R*-factor *wR* and goodness of fit *S* are based on *F*^2^, conventional *R*-factors *R* are based on *F*, with *F* set to zero for negative *F*^2^. The threshold expression of *F*^2^ > σ(*F*^2^) is used only for calculating *R*-factors(gt) *etc*. and is not relevant to the choice of reflections for refinement. *R*-factors based on *F*^2^ are statistically about twice as large as those based on *F*, and *R*- factors based on ALL data will be even larger.

### Fractional atomic coordinates and isotropic or equivalent isotropic displacement parameters (Å^2^)

**Table:**

	*x*	*y*	*z*	*U*_iso_*/*U*_eq_	
N1	0.61026 (7)	0.42994 (12)	0.40175 (8)	0.0224 (4)	
N2	0.48535 (6)	0.36138 (12)	0.46057 (8)	0.0234 (4)	
N3	0.42988 (6)	0.41191 (12)	0.44402 (8)	0.0230 (4)	
N4	0.75138 (7)	0.44728 (13)	0.37188 (9)	0.0250 (4)	
N5	0.77596 (7)	0.53493 (13)	0.34311 (9)	0.0253 (4)	
C1	0.65304 (8)	0.23302 (16)	0.46965 (11)	0.0276 (5)	
H1	0.6677	0.1664	0.4931	0.033*	
C2	0.68692 (8)	0.29596 (15)	0.43668 (10)	0.0260 (4)	
H2	0.7249	0.2723	0.4358	0.031*	
C3	0.66450 (8)	0.39407 (15)	0.40490 (10)	0.0218 (4)	
C4	0.59769 (8)	0.26889 (15)	0.46784 (10)	0.0246 (4)	
H4	0.5735	0.2273	0.4900	0.029*	
C5	0.57755 (8)	0.36729 (15)	0.43294 (10)	0.0214 (4)	
C6	0.51856 (8)	0.40877 (15)	0.42733 (10)	0.0219 (4)	
H6	0.5048	0.4718	0.3986	0.026*	
C7	0.39333 (8)	0.36647 (14)	0.47450 (10)	0.0207 (4)	
C8	0.40803 (7)	0.26922 (14)	0.52396 (10)	0.0204 (4)	
C9	0.40873 (8)	0.27596 (16)	0.59825 (10)	0.0254 (4)	
H9	0.3990	0.3423	0.6174	0.031*	
C10	0.42363 (8)	0.18627 (17)	0.64447 (11)	0.0307 (5)	
H10	0.4245	0.1918	0.6953	0.037*	
C11	0.43716 (9)	0.08927 (16)	0.61696 (11)	0.0303 (5)	
H11	0.4474	0.0280	0.6487	0.036*	
C12	0.43578 (8)	0.08137 (16)	0.54286 (11)	0.0279 (5)	
H12	0.4445	0.0142	0.5236	0.033*	
C13	0.42185 (8)	0.17068 (15)	0.49684 (11)	0.0249 (4)	
H13	0.4217	0.1649	0.4463	0.030*	
C14	0.33326 (8)	0.41441 (14)	0.45463 (10)	0.0209 (4)	
C15	0.32071 (8)	0.50628 (15)	0.40943 (10)	0.0256 (4)	
H15	0.3512	0.5399	0.3939	0.031*	
C16	0.26442 (9)	0.54899 (16)	0.38698 (11)	0.0287 (5)	
H16	0.2564	0.6111	0.3558	0.034*	
C17	0.21974 (9)	0.50112 (16)	0.40998 (11)	0.0289 (5)	
H17	0.1810	0.5301	0.3942	0.035*	
C18	0.23155 (8)	0.41164 (16)	0.45561 (12)	0.0299 (5)	
H18	0.2010	0.3796	0.4720	0.036*	
C19	0.28792 (8)	0.36812 (15)	0.47775 (11)	0.0265 (4)	
H19	0.2957	0.3061	0.5090	0.032*	
C20	0.69872 (8)	0.46825 (15)	0.37254 (10)	0.0239 (4)	
H20	0.6811	0.5343	0.3513	0.029*	
C21	0.82136 (8)	0.51081 (15)	0.32125 (9)	0.0209 (4)	
C22	0.84387 (8)	0.39924 (15)	0.31789 (10)	0.0211 (4)	
C23	0.90133 (8)	0.37120 (16)	0.35746 (10)	0.0267 (4)	
H23	0.9265	0.4241	0.3873	0.032*	
C24	0.92223 (9)	0.26719 (16)	0.35387 (11)	0.0313 (5)	
H24	0.9611	0.2484	0.3820	0.038*	
C25	0.88609 (9)	0.19091 (17)	0.30904 (12)	0.0339 (5)	
H25	0.9004	0.1197	0.3059	0.041*	
C26	0.82924 (9)	0.21799 (16)	0.26881 (12)	0.0340 (5)	
H26	0.8046	0.1655	0.2378	0.041*	
C27	0.80817 (8)	0.32135 (16)	0.27363 (11)	0.0277 (5)	
H27	0.7689	0.3392	0.2464	0.033*	
C28	0.85117 (8)	0.60294 (15)	0.29616 (10)	0.0220 (4)	
C29	0.83879 (8)	0.71002 (15)	0.31126 (10)	0.0246 (4)	
H29	0.8104	0.7240	0.3370	0.030*	
C30	0.86733 (9)	0.79540 (16)	0.28925 (10)	0.0282 (5)	
H30	0.8583	0.8676	0.2997	0.034*	
C31	0.90906 (9)	0.77644 (17)	0.25204 (10)	0.0309 (5)	
H31	0.9294	0.8353	0.2380	0.037*	
C32	0.92088 (9)	0.67150 (17)	0.23551 (11)	0.0318 (5)	
H32	0.9488	0.6583	0.2089	0.038*	
C33	0.89241 (8)	0.58532 (16)	0.25738 (10)	0.0273 (4)	
H33	0.9011	0.5134	0.2458	0.033*	

### Atomic displacement parameters (Å^2^)

**Table:**

	*U*^11^	*U*^22^	*U*^33^	*U*^12^	*U*^13^	*U*^23^
N1	0.0201 (8)	0.0261 (9)	0.0225 (8)	0.0002 (7)	0.0088 (7)	0.0003 (7)
N2	0.0173 (8)	0.0262 (9)	0.0276 (9)	0.0014 (7)	0.0079 (7)	−0.0006 (7)
N3	0.0173 (8)	0.0247 (9)	0.0277 (9)	0.0011 (7)	0.0075 (7)	−0.0011 (7)
N4	0.0230 (9)	0.0262 (9)	0.0297 (9)	0.0000 (7)	0.0136 (7)	0.0020 (7)
N5	0.0226 (9)	0.0271 (9)	0.0296 (9)	−0.0010 (7)	0.0128 (7)	0.0028 (7)
C1	0.0253 (11)	0.0254 (11)	0.0325 (11)	0.0033 (9)	0.0090 (9)	0.0058 (9)
C2	0.0201 (10)	0.0312 (11)	0.0286 (10)	0.0038 (8)	0.0102 (8)	0.0016 (8)
C3	0.0182 (10)	0.0273 (11)	0.0213 (9)	0.0016 (8)	0.0078 (8)	−0.0014 (8)
C4	0.0223 (10)	0.0266 (11)	0.0275 (10)	0.0002 (8)	0.0114 (8)	0.0030 (8)
C5	0.0194 (10)	0.0257 (10)	0.0201 (9)	−0.0015 (8)	0.0070 (8)	−0.0018 (8)
C6	0.0204 (10)	0.0234 (10)	0.0227 (10)	0.0007 (8)	0.0076 (8)	0.0010 (8)
C7	0.0190 (9)	0.0208 (10)	0.0231 (10)	−0.0029 (8)	0.0072 (8)	−0.0041 (8)
C8	0.0128 (9)	0.0237 (10)	0.0255 (10)	−0.0016 (7)	0.0069 (8)	0.0000 (8)
C9	0.0220 (10)	0.0277 (11)	0.0297 (11)	0.0039 (8)	0.0124 (8)	−0.0004 (8)
C10	0.0286 (11)	0.0417 (13)	0.0264 (11)	0.0061 (10)	0.0153 (9)	0.0065 (9)
C11	0.0279 (11)	0.0315 (12)	0.0351 (12)	0.0041 (9)	0.0148 (9)	0.0103 (9)
C12	0.0249 (11)	0.0228 (11)	0.0381 (12)	0.0023 (8)	0.0127 (9)	0.0003 (9)
C13	0.0215 (10)	0.0278 (11)	0.0258 (10)	−0.0007 (8)	0.0073 (8)	−0.0014 (8)
C14	0.0210 (10)	0.0197 (10)	0.0227 (10)	0.0008 (8)	0.0073 (8)	−0.0038 (8)
C15	0.0224 (10)	0.0270 (11)	0.0295 (10)	0.0004 (8)	0.0107 (8)	0.0021 (8)
C16	0.0284 (11)	0.0256 (11)	0.0318 (11)	0.0052 (9)	0.0083 (9)	0.0050 (9)
C17	0.0185 (10)	0.0296 (11)	0.0370 (12)	0.0050 (8)	0.0054 (9)	−0.0023 (9)
C18	0.0182 (10)	0.0277 (11)	0.0462 (13)	−0.0004 (8)	0.0130 (9)	0.0020 (9)
C19	0.0197 (10)	0.0235 (10)	0.0366 (11)	−0.0006 (8)	0.0085 (9)	0.0037 (9)
C20	0.0216 (10)	0.0265 (10)	0.0261 (10)	0.0032 (8)	0.0110 (8)	0.0015 (8)
C21	0.0168 (9)	0.0275 (10)	0.0188 (9)	−0.0010 (8)	0.0059 (7)	−0.0007 (8)
C22	0.0199 (10)	0.0249 (10)	0.0218 (9)	−0.0005 (8)	0.0115 (8)	0.0008 (8)
C23	0.0241 (10)	0.0328 (11)	0.0249 (10)	0.0009 (9)	0.0096 (8)	−0.0005 (8)
C24	0.0269 (11)	0.0374 (12)	0.0326 (11)	0.0109 (9)	0.0135 (9)	0.0087 (9)
C25	0.0422 (13)	0.0255 (11)	0.0448 (13)	0.0059 (10)	0.0297 (11)	0.0063 (10)
C26	0.0335 (12)	0.0293 (12)	0.0456 (13)	−0.0081 (9)	0.0218 (10)	−0.0095 (10)
C27	0.0199 (10)	0.0310 (11)	0.0344 (11)	−0.0040 (9)	0.0113 (9)	−0.0047 (9)
C28	0.0167 (9)	0.0285 (11)	0.0202 (9)	−0.0028 (8)	0.0043 (8)	−0.0005 (8)
C29	0.0220 (10)	0.0293 (11)	0.0221 (10)	0.0007 (8)	0.0057 (8)	0.0007 (8)
C30	0.0311 (11)	0.0255 (11)	0.0245 (10)	−0.0009 (9)	0.0026 (9)	0.0023 (8)
C31	0.0303 (11)	0.0359 (12)	0.0248 (10)	−0.0098 (9)	0.0050 (9)	0.0050 (9)
C32	0.0283 (11)	0.0408 (13)	0.0300 (11)	−0.0060 (9)	0.0140 (9)	−0.0004 (9)
C33	0.0248 (11)	0.0303 (11)	0.0289 (11)	−0.0018 (9)	0.0111 (9)	−0.0022 (9)

### Geometric parameters (Å, °)

**Table:**

N1—C5	1.343 (2)	C15—H15	0.95
N1—C3	1.345 (2)	C16—C17	1.385 (3)
N2—C6	1.278 (2)	C16—H16	0.95
N2—N3	1.407 (2)	C17—C18	1.376 (3)
N3—C7	1.295 (2)	C17—H17	0.95
N4—C20	1.278 (2)	C18—C19	1.388 (3)
N4—N5	1.408 (2)	C18—H18	0.95
N5—C21	1.292 (2)	C19—H19	0.95
C1—C4	1.376 (3)	C20—H20	0.95
C1—C2	1.385 (3)	C21—C22	1.485 (3)
C1—H1	0.95	C21—C28	1.485 (3)
C2—C3	1.386 (3)	C22—C27	1.387 (3)
C2—H2	0.95	C22—C23	1.394 (3)
C3—C20	1.466 (3)	C23—C24	1.385 (3)
C4—C5	1.397 (3)	C23—H23	0.95
C4—H4	0.95	C24—C25	1.383 (3)
C5—C6	1.464 (2)	C24—H24	0.95
C6—H6	0.95	C25—C26	1.383 (3)
C7—C14	1.487 (2)	C25—H25	0.95
C7—C8	1.496 (2)	C26—C27	1.383 (3)
C8—C9	1.391 (3)	C26—H26	0.95
C8—C13	1.393 (3)	C27—H27	0.95
C9—C10	1.388 (3)	C28—C33	1.393 (3)
C9—H9	0.95	C28—C29	1.400 (3)
C10—C11	1.378 (3)	C29—C30	1.378 (3)
C10—H10	0.95	C29—H29	0.95
C11—C12	1.384 (3)	C30—C31	1.385 (3)
C11—H11	0.95	C30—H30	0.95
C12—C13	1.380 (3)	C31—C32	1.379 (3)
C12—H12	0.95	C31—H31	0.95
C13—H13	0.95	C32—C33	1.384 (3)
C14—C19	1.392 (3)	C32—H32	0.95
C14—C15	1.396 (3)	C33—H33	0.95
C15—C16	1.383 (3)		
			
C5—N1—C3	117.13 (16)	C17—C16—H16	120.0
C6—N2—N3	110.94 (15)	C18—C17—C16	119.95 (18)
C7—N3—N2	114.33 (15)	C18—C17—H17	120.0
C20—N4—N5	111.17 (15)	C16—C17—H17	120.0
C21—N5—N4	114.78 (15)	C17—C18—C19	120.17 (19)
C4—C1—C2	118.69 (18)	C17—C18—H18	119.9
C4—C1—H1	120.7	C19—C18—H18	119.9
C2—C1—H1	120.7	C18—C19—C14	120.72 (18)
C1—C2—C3	118.84 (18)	C18—C19—H19	119.6
C1—C2—H2	120.6	C14—C19—H19	119.6
C3—C2—H2	120.6	N4—C20—C3	122.41 (17)
N1—C3—C2	123.35 (17)	N4—C20—H20	118.8
N1—C3—C20	114.35 (16)	C3—C20—H20	118.8
C2—C3—C20	122.30 (17)	N5—C21—C22	124.72 (17)
C1—C4—C5	119.08 (18)	N5—C21—C28	116.10 (16)
C1—C4—H4	120.5	C22—C21—C28	119.16 (16)
C5—C4—H4	120.5	C27—C22—C23	118.72 (18)
N1—C5—C4	122.86 (17)	C27—C22—C21	120.22 (17)
N1—C5—C6	115.26 (16)	C23—C22—C21	121.04 (17)
C4—C5—C6	121.88 (17)	C24—C23—C22	120.84 (19)
N2—C6—C5	120.97 (17)	C24—C23—H23	119.6
N2—C6—H6	119.5	C22—C23—H23	119.6
C5—C6—H6	119.5	C25—C24—C23	119.56 (19)
N3—C7—C14	115.53 (16)	C25—C24—H24	120.2
N3—C7—C8	123.75 (16)	C23—C24—H24	120.2
C14—C7—C8	120.66 (15)	C26—C25—C24	120.19 (19)
C9—C8—C13	118.80 (17)	C26—C25—H25	119.9
C9—C8—C7	120.45 (16)	C24—C25—H25	119.9
C13—C8—C7	120.75 (16)	C25—C26—C27	120.07 (19)
C10—C9—C8	120.36 (18)	C25—C26—H26	120.0
C10—C9—H9	119.8	C27—C26—H26	120.0
C8—C9—H9	119.8	C26—C27—C22	120.60 (19)
C11—C10—C9	120.28 (18)	C26—C27—H27	119.7
C11—C10—H10	119.9	C22—C27—H27	119.7
C9—C10—H10	119.9	C33—C28—C29	118.18 (17)
C10—C11—C12	119.77 (18)	C33—C28—C21	121.01 (17)
C10—C11—H11	120.1	C29—C28—C21	120.81 (17)
C12—C11—H11	120.1	C30—C29—C28	120.78 (18)
C13—C12—C11	120.26 (18)	C30—C29—H29	119.6
C13—C12—H12	119.9	C28—C29—H29	119.6
C11—C12—H12	119.9	C29—C30—C31	120.34 (19)
C12—C13—C8	120.52 (18)	C29—C30—H30	119.8
C12—C13—H13	119.7	C31—C30—H30	119.8
C8—C13—H13	119.7	C32—C31—C30	119.52 (19)
C19—C14—C15	118.32 (17)	C32—C31—H31	120.2
C19—C14—C7	121.87 (16)	C30—C31—H31	120.2
C15—C14—C7	119.78 (17)	C31—C32—C33	120.49 (19)
C16—C15—C14	120.84 (18)	C31—C32—H32	119.8
C16—C15—H15	119.6	C33—C32—H32	119.8
C14—C15—H15	119.6	C32—C33—C28	120.67 (19)
C15—C16—C17	119.98 (18)	C32—C33—H33	119.7
C15—C16—H16	120.0	C28—C33—H33	119.7
